# Effects of Wood Vinegar as a Coagulant in Rubber Sheet Production: A Sustainable Alternative to Acetic Acid and Formic Acid

**DOI:** 10.3390/polym17131718

**Published:** 2025-06-20

**Authors:** Visit Eakvanich, Putipong Lakachaiworakun, Natworapol Rachsiriwatcharabul, Wassachol Wattana, Wachara Kalasee, Panya Dangwilailux

**Affiliations:** 1 Department of Engineering, King Mongkut’s Institute of Technology Ladkrabang, Chumphon Campus, Chumphon 86160, Thailand; visit.ea@kmitl.ac.th (V.E.); wassachol.wa@kmitl.ac.th (W.W.); wachara.ka@kmitl.ac.th (W.K.); 2 Department of Sustainable Industrial Management Engineering, Faculty of Engineering, Rajamangala University of Technology Phra Nakhon, Bangkok 10800, Thailand; putipong.l@rmutp.ac.th (P.L.); natworapol.r@rmutp.ac.th (N.R.)

**Keywords:** ribbed smoked sheets (RSSs), wood vinegar, equilibrium moisture content, sorption isotherm, drying kinetics

## Abstract

Occupational exposure to commercial formic and acetic acids through dermal contact and inhalation during rubber sheet processing poses significant health risks to workers. Additionally, the use of these acids contributes to environmental pollution by contaminating water sources and soil. This study investigates the potential of three types of wood vinegar—derived from para-rubber wood, bamboo, and eucalyptus—obtained through biomass pyrolysis under anaerobic conditions, as sustainable alternatives to formic and acetic acids in the production of ribbed smoked sheets (RSSs). The organic constituents of each wood vinegar were characterized using gas chromatography and subsequently mixed with fresh natural latex to produce coagulated rubber sheets. The physical and chemical properties, equilibrium moisture content, and drying kinetics of the resulting sheets were then evaluated. The results indicated that wood vinegar derived from para-rubber wood contained a higher concentration of acetic acid compared to that obtained from bamboo and eucalyptus. As a result, rubber sheets coagulated with para-rubber wood and bamboo vinegars exhibited moisture sorption isotherms comparable to those of sheets coagulated with acetic acid, best described by the modified Henderson model. In contrast, sheets coagulated with eucalyptus-derived vinegar and formic acid followed the Oswin model. In terms of physical and chemical properties, extended drying times led to improved tensile strength in all samples. No statistically significant differences in tensile strength were observed between the experimental and reference samples. The concentration of acid was found to influence Mooney viscosity, the plasticity retention index (PRI), the thermogravimetric curve, and the overall coagulation process more significantly than the acid type. The drying kinetics of all five rubber sheet samples displayed similar trends, with the drying time decreasing in response to increases in drying temperature and airflow velocity.

## 1. Introduction

Environmental pollution represents a significant global challenge, requiring coordinated efforts across all sectors to develop and implement effective mitigation strategies [1]. The ribbed smoked sheet (RSS) rubber production industry is a major contributor to environmental degradation, particularly through air pollution associated with the drying process, which relies on the combustion of para rubber-wood fuel [1,2,3]. This process emits particulate soot, smoke, and polycyclic aromatic hydrocarbons (PAHs). Furthermore, water and odor pollution arise during the coagulation of fresh latex, a process facilitated by the addition of formic or acetic acid. The use of these acids leads to water contamination, and in the absence of adequate wastewater treatment facilities at RSS production plants, acidic wastewater may pollute public water resources. Additionally, the acid-mixing process poses significant health risks to workers, primarily through the inhalation of emitted odors and direct contact with the acids via the skin [4].

In Thailand, the production of ribbed smoked sheets (RSSs) is predominantly carried out by more than 700 small-scale rubber farmer cooperatives, primarily located in the southern, eastern, northeastern, and northern regions of the country [4,5,6]. The production process begins with farmers tapping rubber trees to collect fresh latex, which is then transported to cooperatives where they hold membership. At the cooperative facilities, the latex is filtered to remove impurities. The purified latex is subsequently mixed with water and either formic acid or acetic acid in appropriate proportions. The mixture is thoroughly stirred to ensure homogeneity, and any foam generated is skimmed off. The latex is then poured into molds equipped with dividers and allowed to coagulate for approximately 3–4 h. Once coagulation is complete, the dividers are removed, and the coagulated latex sheets are transferred to a conveyor system leading to rolling machines. During the rolling process, water is used to lubricate the rollers. Following the rolling process, the raw rubber sheets are hung on bamboo racks to air dry overnight. Subsequently, the sheets are moved to metal racks and subjected to a smoking process in a chamber for approximately 4–5 days, resulting in the final production of ribbed smoked sheets [4,7,8,9]. The wastewater generated during this production process contains residual formic acid or acetic acid, along with water used to clean acid-contaminated equipment and containers before, during, and after the production stages [1,4,9].

Wood vinegar is a renewable bio-product derived from the pyrolytic combustion of biomass [10,11,12]. This liquid is produced through the condensation of smoke during the pyrolysis process, which occurs under anaerobic conditions at temperatures ranging from 300 °C to 400 °C [10,13,14]. The thermal decomposition of various compounds in the biomass leads to the formation of a brownish-red, acidic liquid. The primary constituents of wood vinegar include approximately 85% water, 3% organic acids, and 12% other organic compounds [11,15]. The liquid typically has a pH ranging from 1.5 to 3.0 and a specific gravity between 1.012 and 1.024, with variations depending on the type of biomass used [14,16,17,18]. Furthermore, wood vinegar contains over 200 distinct organic compounds generated during the thermal degradation of biomass, including organic acids and alcohols derived from the breakdown of lignin [12,15]. Notable components include acetic acid, phenolic compounds, and ethyl valerate [12,14].

The equilibrium moisture content refers to the minimum moisture level of a material at which water can continue to evaporate under specific ambient humidity and temperature conditions [19]. This parameter is regarded as a critical factor in developing mathematical models for predicting the moisture content of materials during various drying processes [19,20]. These models are particularly relevant for agricultural materials, which often contain water levels ranging from 50% to 80% of their total weight. Factors such as material type, porosity, and cell structure significantly influence moisture content and are essential considerations in the design and construction of efficient drying systems [4,5,21]. A comprehensive review of the literature identifies several widely utilized mathematical models for analyzing and predicting the moisture content of agricultural materials [21,22]. These models include Oswin, Smith, Henderson, modified Oswin, modified Halsay, and modified Henderson. Despite significant advancements in this field, research on the equilibrium moisture content of rubber sheets remains limited. Existing mathematical models primarily focus on analyzing and predicting the moisture contents of other agricultural materials, with limited applicability to rubber sheets. Moisture reduction in rubber sheets, aimed at producing air-dried products, is currently achieved through two primary methods: sun drying and hot-air drying [22,23]. Studies on sun drying have shown that drying durations vary according to climatic conditions, with factors such as cloud cover and rainfall contributing to fluctuations in solar heat throughout the day [22]. These variations result in slower drying rates and an increased risk of mold growth on the rubber sheets. In contrast, hot-air drying significantly reduces the drying time by 10–12 days compared to sun drying [4,22]. Additionally, comparative analyses indicate that rubber sheets dried using hot air exhibit superior physical properties, attributed to the ability to maintain consistent drying temperatures. While existing research provides valuable insights into moisture reduction and drying times for rubber sheets, no studies have yet addressed the prediction of these parameters for rubber sheets produced by incorporating wood vinegar into fresh latex as a coagulant to replace formic acid and acetic acid. This knowledge gap presents a substantial challenge in the design and development of efficient ribbed smoked sheet production processes. Innovations in this area are essential for reducing energy consumption, decreasing drying times, mitigating product damage, and promoting environmental sustainability. Therefore, the objective of this research is to compare the chemical and physical properties of rubber sheets produced using wood vinegar as a coagulant with those produced using conventional commercial coagulants, such as formic acid and acetic acid. Furthermore, this study seeks to develop mathematical models capable of predicting the EMC of rubber sheets. The findings will contribute to the design and development of optimized drying chambers for RSS production in the future.

## 2. Materials and Methods

### 2.1. Raw Materials

In this study, fresh latex derived from RRIM 600 rubber trees (Hevea brasiliensis) was utilized. The latex was sourced from the Agriculture, Food, and Energy Center of King Mongkut’s Institute of Technology Ladkrabang, Chumphon Campus, Pathio District, Chumphon Province, Thailand. The total solid content (TSC) of fresh latex was determined using the moisture evaporation method (D1076:1988) [24], as specified by the American Society for Testing and Materials (ASTM). For the determination of the dry rubber content (DRC), a 5% (*v*/*v*) acetic acid solution was employed to coagulate the fresh latex. Upon completion of these procedures, the TSC and DRC of the fresh latex used in this study were found to be 31.7 ± 0.8% and 18.2 ± 0.5%, respectively.

### 2.2. Wood Vinegars and Commercial Acid

**Figure 1:**
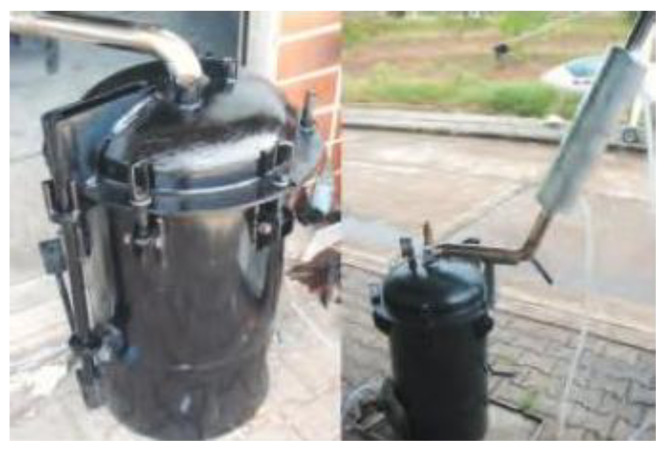
A charcoal kiln and wood vinegar generator.

In this study, unpurified wood vinegar derived from para-rubber wood, eucalyptus wood, and bamboo was utilized as a substitute for formic acid and acetic acid in the production of RSS. The wood vinegar was produced using a system that was previously designed and developed by the research team. In this section, wood vinegar was produced by loading 50 kg of raw biomass—para-rubber wood, bamboo, or eucalyptus—into a charcoal kiln. The biomass was ignited and pyrolyzed separately by type. The kiln’s smoke outlet, constructed from stainless steel, was fitted with an internal water-cooled condenser and insulated externally. The condensed wood vinegar was collected in a storage container positioned beneath the outlet, as illustrated in Figure 1. The resulting wood vinegar exhibited a dark yellow color, and its pH was measured using a pH meter (Mettler Toledo, Greifensee, Switzerland). The wood vinegar was then stored in opaque containers for further analysis. Its chemical composition was analyzed using gas chromatography (Hewlett-Packard 5890 Series II, Waldbronn, Germany). In contrast, formic acid and acetic acid were sourced from commercial suppliers in Thailand.

### 2.3. Hot-Air Drying Chamber

**Figure 2:**
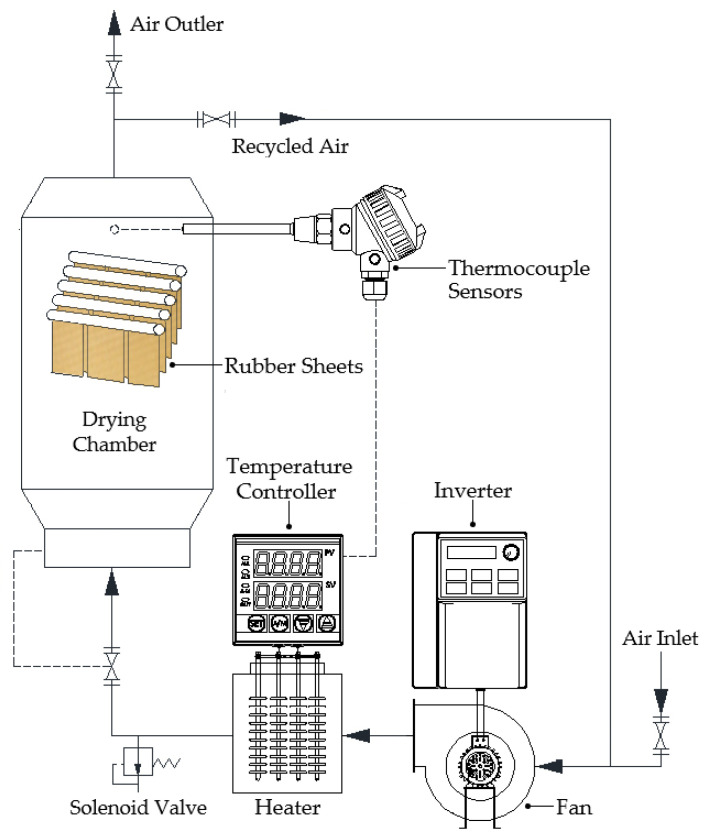
Schematic diagram of the drying system.

The hot-air drying chamber utilized in this study is illustrated in Figure 2. The chamber, constructed with a steel frame, was designed with dimensions of 600 mm in width, 800 mm in length, and 1000 mm in height. A 1.5 kW heater (Maxthermo, model MC-2438, Taipei Hsien, Taiwan) was installed at the base of the unit, along with a centrifugal fan (Nitco, model RB60-520, Hessdorf, Germany) powered by a 2.2 kW motor. These components were used to control the temperature and airflow velocity, ensuring precise regulation during operation.

### 2.4. Preparation of Raw and Dried Rubber Sheets for Testing

The preparation of raw and dried rubber sheets for testing involves the addition of a coagulant to diluted fresh latex (filtered fresh latex, 2000 mL + water, 3000 mL). During the coagulation process, the latex completely coagulates within three hours when treated with various types of wood vinegar or commercial formic acid and acetic acid. The coagulated rubber sheets are subsequently rolled to a thickness of 0.3 cm, washed with clean water, and air dried for 12 h to achieve an initial moisture content of approximately 50%. The product obtained through this process is referred to as raw rubber sheets. For testing, a portion of the raw rubber sheets is analyzed to determine their physical and chemical properties, equilibrium moisture content, and drying kinetics. The remaining sheets are dried at 60 °C for 48 h to produce dried rubber sheets with a final moisture content of approximately 3.0%. These dried rubber sheets are then analyzed to evaluate their physical and chemical properties.

### 2.5. Properties of Rubber Sheets

The properties of the rubber sheets were assessed by testing the dirt content [25], volatile matter content [26], Mooney viscosity [27], and plasticity retention index (PRI) [28] for both raw and dried rubber sheets, following the Standard Malaysian Rubber (SMR) methods. The vulcanization process, including the cure time (t90) [ACS#1 formula, ODR type, TECH PRO, Arc 1.5 at 150 °C], was conducted in accordance with the International Organization for Standardization (ISO) method (ISO 3417–1977) [29]. Furthermore, the mechanical properties of the vulcanized rubber sheets were evaluated by tensile strength testing (ISO 37 Type 1) [30].

### 2.6. Determination and Analysis of the Equilibrium Moisture Content of Rubber Sheets

**Figure 3:**
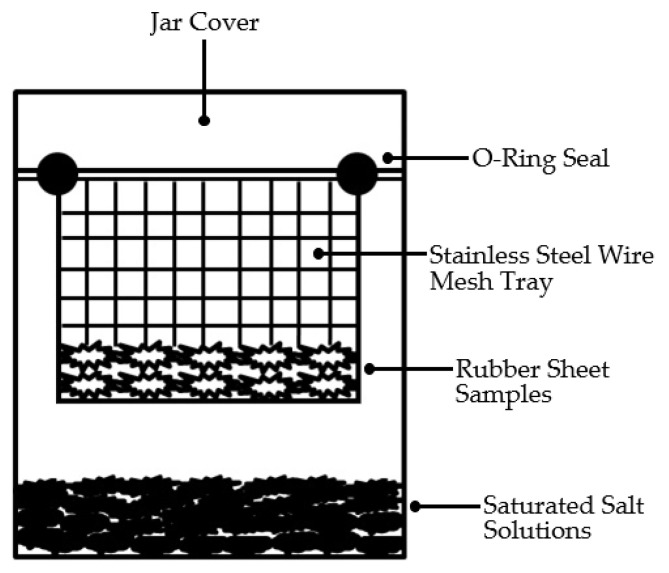
Setup glass jar vessel for equilibrium moisture content determination.

The equilibrium moisture content (EMC) of the raw rubber sheets in this study was determined and analyzed using the static desiccator isotherm method for moisture adsorption, a statistical approach. Saturated salt solutions of six salts—lithium chloride (LiCl), magnesium chloride (MgCl_2_), magnesium nitrate (Mg(NO₃)_2_), potassium iodide (KI), sodium chloride (NaCl), and diammonium sulfate ((NH₄)_2_SO₄)—with water activity (aw) values ranging from 0.108 to 0.799 were employed at temperatures of 40, 50, and 60 °C, respectively, as detailed in Table 1. The equipment utilized to test the EMC of the raw rubber sheets consisted of six 750-milliliter jars, each containing 200 milliliters of one of the saturated salt solutions. To prepare the rubber sheets, fresh latex was coagulated using wood vinegar (derived from rubberwood, bamboo, and eucalyptus), formic acid, and acetic acid. Five types of rubber sheets were cut into six pieces, with each piece weighing approximately 10 g. These pieces were further cut into 2 × 2 cm sections and placed on mesh trays, ensuring that the sheets did not overlap. The mesh trays holding the rubber sheet samples were then positioned in the jars, ensuring that the mesh did not come into contact with the saturated salt solutions. The jars were tightly sealed, as illustrated in Figure 3, and placed in an oven with controlled temperature settings to evaluate the moisture release from each type of rubber sheet at 40, 50, and 60 °C. Once the weight of each sample stabilized (with a variation of less than ±0.001 g), the rubber sheets were removed from the jars and dried at 103 °C for 24 h [31] to determine their dry weight. The EMC was subsequently calculated using Equation (1).

(1) $$\mathrm{EMC}=\frac{m_{\mathrm{eq}}-m_{f}}{m_{f}}$$

where EMC, m_eq_, and m_f_ are the equilibrium moisture content of the rubber sheet in dry basis percentage, the weight of the rubber sheet at equilibrium, and the dry weight of the rubber sheet

### 2.7. Determination of Sorption Isotherm Model

In this study, six isotherm adsorption models were employed to predict the equilibrium moisture contents (EMCs) of five types of raw rubber sheets. These sheets were coagulated using wood vinegar derived from para-rubber wood, eucalyptus, and bamboo, as well as acetic acid and formic acid. This study aimed to establish correlations between the experimental data, ambient temperature (45–60 °C), and relative humidity (10–97%). The six EMC models were classified into two categories: Type I models, which describe the relationship between moisture content and ambient relative humidity, and Type II models, which represent EMC as a function of both ambient relative humidity and temperature. The EMC models utilized in this study are presented in Equations (2) to (7) in Table 2.

### 2.8. Drying Kinetics Testing

In this study, raw rubber sheet samples were placed in a drying chamber, where the drying temperature was set at 40, 50, and 60 °C, respectively. The inlet air velocity was maintained at 0.5 m/s and 1.0 m/s, based on data obtained from drying experiments and computational fluid dynamics (CFD) simulations of rubber sheet drying [4,5,7,19]. The initial moisture content of the rubber sheets was on the dry basis, while the final moisture content was maintained below 3.0% [4,21,22]. The ambient air temperature, along with the inlet and outlet drying air temperatures, was measured using K-type thermocouples. The moisture content of the natural rubber sheet samples was determined following the standard method of the Association of Official Analytical Chemists [31].

### 2.9. Thermogravimetric Analysis (TGA)

Thermal stability of the samples was evaluated using a thermogravimetric analyzer (TGA, PerkinElmer, Shelton, Connecticut, CT, USA). Approximately 7–10 mg of each sample was placed in a crucible and heated from 40 °C to 800 °C at a constant rate of 10 °C/min under a nitrogen atmosphere, with a flow rate of 10 mL/min.

### 2.10. Statistical Analysis

Analysis of variance (ANOVA) was utilized for graphical data analysis to identify interactions between the responses and to estimate statistical parameters and process variables. Key model conditions were evaluated using *p*-values at a 95% confidence level.

## 3. Results and Discussion

### 3.1. Chemical Composition of Wood Vinegar

Wood vinegar derived from para-rubber wood, bamboo, and eucalyptus was analyzed, with pH values of 3.77, 3.64, and 3.52, respectively. The analysis identified five major chemical components: acetic acid, phenol, syringol (phenol, 2,6-dimethoxy), guaiacol (2-methoxyphenol), and p-cresol, as summarized in Table 3. Among these, acetic acid emerged as the predominant organic compound, accounting for approximately 80% of the total organic content in all three types of wood vinegar. The highest acetic acid concentration was observed in rubber wood-derived vinegar, followed by bamboo- and eucalyptus-derived vinegar. These chemical compounds contribute to the distinct pungent odor of wood vinegar. Acetic acid is responsible for its sour aroma, while guaiacol, p-cresol, and syringol impart a smoky scent [38,39]. The organic composition of wood vinegar is consistent across the three sources, resulting in similar olfactory profiles. Furthermore, the chemical composition serves as a key indicator of wood vinegar quality. High-quality wood vinegar is characterized by elevated levels of acetic acid and phenolic compounds, which exhibit strong antifungal properties, effectively inhibiting the growth of pathogenic fungi [4,18].

The research findings presented in Table 3 indicate that the chemical composition of the three types of wood vinegar varies in both concentration and component type, likely due to differences in the wood sources used for production. The primary components of wood vinegar are carbonization products derived from lignin, a major constituent of lignocellulose [4,38], which consists of phenylpropane units. Among the three types, para-rubber wood vinegar exhibited the highest concentrations of acetic acid and phenolic compounds [4,18], which play a crucial role in coagulating fresh latex into a tofu-like solid and inhibiting fungal growth on rubber sheets. Bamboo wood vinegar contained the second-highest concentrations of these compounds, followed by eucalyptus wood vinegar. Therefore, the chemical composition of wood vinegar produced from biomass charcoal pyrolysis is influenced by the type of wood used. The highest concentration of key compounds was found in para-rubber wood vinegar, suggesting that its production yields a higher-quality wood vinegar compared to that derived from bamboo and eucalyptus, respectively.

### 3.2. Chemical, Physical, and Mechanical Properties of NR Sheets

Table 4 presents the chemical, physical, and mechanical properties of NR sheets, categorized based on five different coagulants: wood vinegar derived from para-rubber wood, bamboo, and eucalyptus, as well as acetic acid and formic acid. The experimental results indicate that the average levels of impurities and volatile substances in NR sheets coagulated with wood vinegar from para-rubber wood, bamboo, and eucalyptus were not significantly different from those coagulated with acetic acid and formic acid. This similarity is attributed to the presence of acetic acid as a primary component of wood vinegar (see Table 3). Regarding the physical properties, Table 4 illustrates the variations in tensile strength at break during the drying process. The results indicate that increasing the drying duration enhances tensile strength at break across all samples. This effect is attributed to the reduction in intermolecular spacing within the rubber matrix as water molecules evaporate [4,18,22,36]. A comparison of tensile strength at break and elongation at break among the five samples showed values ranging from 4.8 ± 0.5 MPa to 6.4 ± 0.3 MPa and from 625 ± 8% to 678 ± 7%, respectively. The natural rubber (NR) sheets coagulated with acetic acid exhibited the highest values for both tensile strength and elongation at break, followed by those treated with wood vinegar derived from para-rubber wood, bamboo-derived wood vinegar, formic acid, and eucalyptus-derived wood vinegar, respectively. Regarding the 300% modulus, the results indicated no statistically significant differences among the samples. Additionally, Table 4 presents the effects of different coagulating agents on the Mooney viscosity and plasticity retention index (PRI) of rubber sheets. A comparison of the phenolic group contents among the five coagulating agents revealed the following order: formic acid > eucalyptus wood-derived vinegar > para-rubber wood-derived vinegar > bamboo wood-derived vinegar > acetic acid. The predominant formation of phenolic groups in NR latex results from reactions involving amine groups in the rubber [18,36], which leads to an increase in the Mooney viscosity and PRI of NR sheets. Therefore, it can be concluded that phenolic groups in NR latex significantly influence the Mooney viscosity and PRI of rubber sheets, consistent with the findings reported in [4,22]. Notably, the results indicate that the acid concentration has a greater impact on Mooney viscosity, PRI, and the manufacturing process than the specific type of acid used.

Before NR can be utilized in production, its molecules must undergo crosslinking to enhance elasticity. Typically, NR products are prepared with 90% crosslinking, and the time required to achieve 90% crosslinking is referred to as the vulcanization or curing time (t90). The t90 values for each type of dried NR do not differ significantly, as shown in Table 4. Note: A previous study by the research team [40] found that the creep and stress relaxation properties of rubber sheets coagulated with wood vinegar derived from all three biomass types did not differ significantly from those of sheets produced using formic acid and acetic acid.

### 3.3. The Experimental Results for the EMC of Rubber Sheets

**Figure 4:**
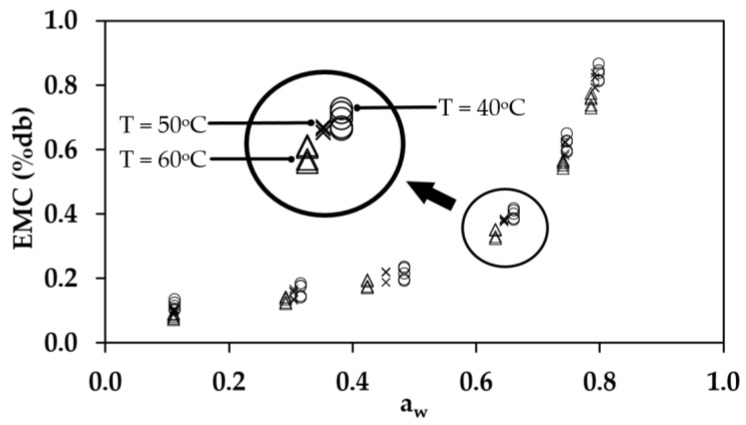
The experimental results for the EMC of rubber sheets at temperatures of 40, 50, and 60 °C within a water activity (a_w_) range of 0.110–0.821.

Figure 4 presents the experimental results for the EMC of rubber sheets at temperatures of 40, 50, and 60 °C within a water activity (a_w_) range of 0.110–0.821. The findings indicate that, at a constant temperature, EMC is dependent on water activity. Specifically, lower water activity values correspond to lower EMC values, whereas higher water activity results in an increased EMC. This trend occurs because, with higher water activity, the relative humidity of the surrounding environment also increases, thereby reducing moisture transfer between the material and its surroundings compared to conditions with lower relative humidity. Furthermore, at a constant water activity level, EMC is influenced by temperature. Lower temperatures result in higher EMC values, whereas increasing the temperature leads to a decrease in EMC. This effect is attributed to the greater molecular mobility of water at elevated temperatures, which weakens intermolecular attraction forces and ultimately reduces moisture retention in the rubber sheets [18,22,36].

### 3.4. The Results for Sorption Isotherm Model

**Figure 5:**
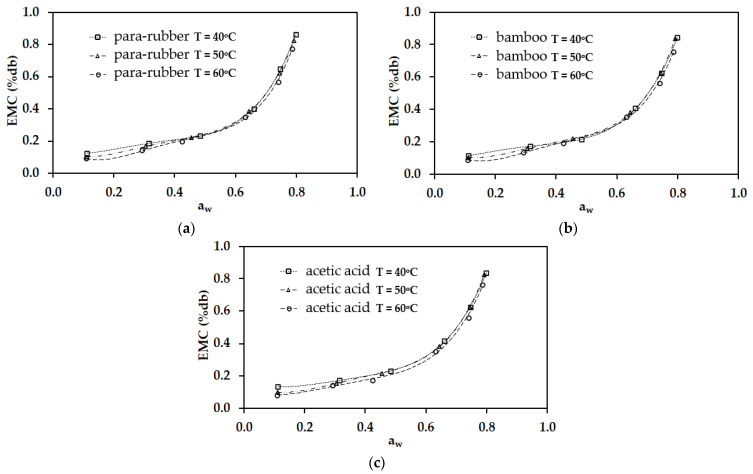
A comparative analysis of the modified Henderson models with the experimental results at each temperature. (**a**) Para-rubber wood-derived vinegar. (**b**) Bamboo-derived vinegar. (**c**) Acetic acid.

**Figure 6:**
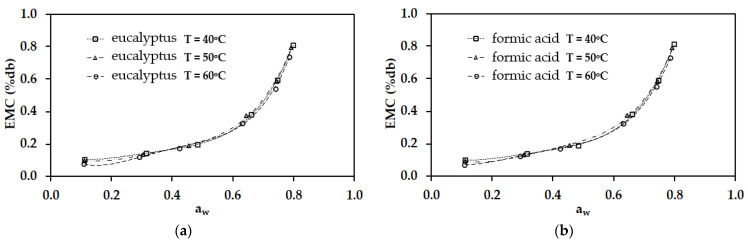
A comparative analysis of the Oswin models with the experimental results at each temperature. (**a**) Eucalyptus wood-derived vinegar. (**b**) Formic acid.

A comparison of six EMC mathematical models with experimental results at each temperature is presented in Table 5 and Table 6. The findings indicate that the modified Henderson model provides the most accurate prediction of EMC for rubber sheets coagulated with para-rubber wood-derived vinegar, bamboo-derived vinegar, and acetic acid, exhibiting only minor deviations from the experimental data. In contrast, for rubber sheets coagulated with eucalyptus-derived vinegar and formic acid, the Oswin model demonstrates closer agreement with the experimental results. This difference can be attributed to the composition of the coagulants. Para-rubber wood-derived and bamboo-derived vinegars contain significantly higher levels of acetic acid than eucalyptus-derived vinegar (see Table 3). As a result, they induce a moisture adsorption process more similar to that of acetic acid than formic acid. Similarly, eucalyptus-derived vinegar contains higher levels of phenolic compounds (see Table 3, items 2–4) and lower levels of acetic acid compared to para-rubber wood-derived and bamboo-derived vinegars. Consequently, the equilibrium moisture modeling characteristics of rubber sheets coagulated with eucalyptus-derived vinegar more closely resemble those of rubber sheets coagulated with formic acid. Additionally, a comparative analysis of the modified Henderson and Oswin models with the experimental results at each temperature is illustrated in Figure 5 and Figure 6, respectively. Figure 5 corresponds to rubber sheets coagulated with para-rubber wood-derived vinegar, bamboo-derived vinegar, and acetic acid, while Figure 6 represents those coagulated with eucalyptus wood-derived vinegar and formic acid.

### 3.5. Drying Kinetics of NR Sheets

**Figure 7:**
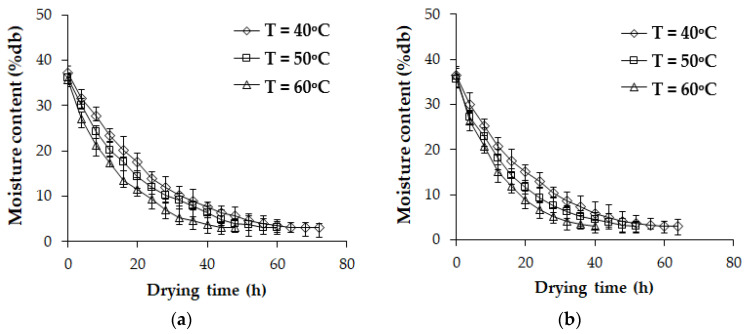
The moisture ratio during the drying of rubber sheets at air temperatures of 40, 50, and 60 °C. (**a**) Airflow velocities of 0.5 m/s. (**b**) Airflow velocities of 1 m/s.

Figure 7 illustrates the moisture ratio during the drying of rubber sheets at air temperatures of 40, 50, and 60 °C with airflow velocities of 0.5 and 1 m/s, respectively. The results indicate that, at a constant airflow velocity, moisture in the rubber sheets evaporates more rapidly at higher temperatures than at lower temperatures. This phenomenon is attributed to increased molecular excitation of water at elevated temperatures, which weakens intermolecular forces and accelerates moisture evaporation [22,36]. Similarly, at a constant temperature, moisture in the rubber sheets evaporates more rapidly at higher airflow velocities than at lower velocities. This occurs because higher airflow velocities enhance moisture removal from the surface of the rubber sheets more effectively than lower velocities.

### 3.6. The Results of the Thermogravimetric Analysis (TGA)

**Figure 8:**
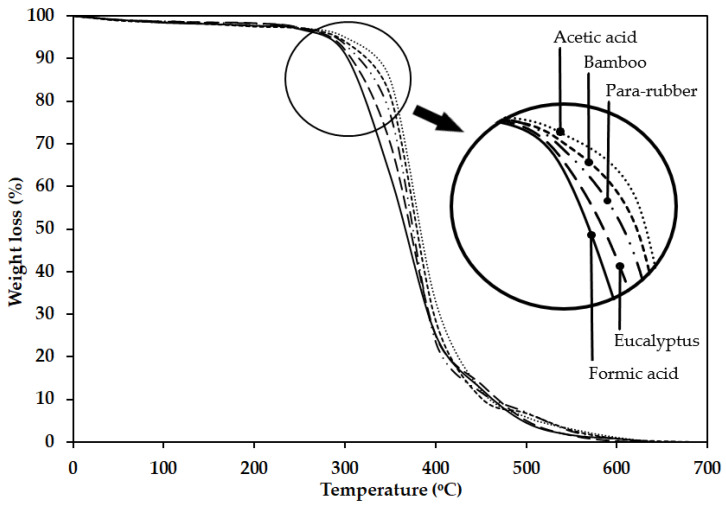
Thermogravimetric analysis curves for rubber sheet samples.

TGA is a widely adopted technique for evaluating the thermal stability of elastomeric composites, including natural rubber. Figure 8 displays the TGA thermograms, which depict the weight loss of rubber sheet samples as a function of temperature and offer insights into their thermal degradation behavior. The samples were coagulated using formic acid, acetic acid, and wood vinegar derived from para-rubber wood, eucalyptus, and bamboo, respectively. The TGA results revealed that the thermal degradation of the rubber sheets occurred in three distinct stages. The first stage, between 285 °C and 345 °C, corresponds to the evaporation of volatile compounds, primarily involving the dehydration of hydroxyl groups followed by the formation of volatile organic compounds. The second stage, which contributed to the greatest weight loss, occurred between 345 °C and 405 °C and was attributed to the decomposition of rubber molecules. During this stage, thermal crosslinking is initiated, followed by the breakdown of the resulting crosslinked structures. Significant weight loss during this phase is associated with the decomposition of major rubber constituents, including isoprene (39%), dipentene (13.2%), and trace amounts of p-methane [36,41]. The third stage, occurring from 405 °C to 650 °C, represents the final degradation phase of the rubber sheets. The thermal stability of the rubber sheets varied according to the type of coagulant used. As shown in Figure 8, the TGA thermograms shifted toward higher decomposition temperatures in the following order: formic acid < eucalyptus wood vinegar < para-rubber wood vinegar < bamboo wood vinegar < acetic acid. This trend indicates that residual acetic acid contributes positively to thermal stability; specifically, higher concentrations of residual acetic acid are associated with increased thermal resistance. The thermal stability patterns observed in the TGA thermograms were consistent with the corresponding t90 values (see Table 4).

## 4. Conclusions

The research findings indicate that para-rubber wood-derived vinegar contains a higher concentration of acetic acid than bamboo-derived and eucalyptus-derived vinegars. Consequently, rubber sheets produced using para-rubber wood- and bamboo-derived vinegars exhibit adsorption isotherm behavior similar to those coagulated with acetic acid, best described by the modified Henderson model. In contrast, eucalyptus-derived vinegar has a higher concentration of phenolic compounds. As a result, rubber sheets produced using eucalyptus-derived vinegar exhibit adsorption isotherm behavior akin to those coagulated with formic acid, following the Oswin model. Regarding physical and chemical properties, tensile strength increased with an extended drying time across all samples. The acid concentration had a greater influence on Mooney viscosity, the plasticity retention index (PRI), the thermogravimetric curve, and the overall production process than the specific type of acid used. The drying kinetics of all five rubber sheet samples exhibited similar trends, with the drying time increasing as the drying temperature and airflow velocity decreased. Recommendations: This study presents an environmentally sustainable approach to mitigating both water and air pollution associated with the production of RSS by rubber farmer cooperatives. Water pollution is addressed through the use of wood vinegar, a byproduct of charcoal production, as a cost-effective alternative to conventional chemical coagulants. Air pollution is reduced by substituting traditional smoke-drying methods with hot-air drying. The rubber sheets produced using this method exhibited no statistically significant differences in mechanical or physicochemical properties compared to those produced by conventional means. Furthermore, the cost of constructing a hot-air drying chamber and producing air-dried rubber sheets via this method is lower than that required for building or upgrading traditional smoke-drying chambers used in conventional production [1,5,6,42].

## Figures and Tables

**Table 1:** Water activity of various saturated salt solutions at 40, 50, 60, and 70 °C.

Salts	Water Activity		
	40 °C	50 °C	60 °C
LiCl	0.112	0.111	0.110
MgCl_2_	0.316	0.305	0.292
Mg(NO_3_)_2_	0.484	0.454	0.424
KI	0.661	0.645	0.631
NaCl	0.747	0.744	0.741
(NH_4_)_2_SO_4_	0.799	0.792	0.786

**Table 2:** Illustration of equilibrium moisture content models.

**Model Type I**		
Oswin [32]	$m_{\mathrm{eq}}=A{\left[a_{w}/(1-a_{w})\right]}^{B}$	(2)
Smith [33]	$m_{\mathrm{eq}}=A-\mathrm{Bln}(1-a_{w})$	(3)
Henderson [34]	$m_{\mathrm{eq}}={\left[-\ln(1-a_{w})/\mathrm{AT}\right]}^{1/B}$	(4)
**Model Type II**		
Modified Oswin [35]	$m_{\mathrm{eq}}=(A+\mathrm{BT}){\left[a_{w}/(1-a_{w})\right]}^{C}$	(5)
Modified Henderson [36]	$m_{\mathrm{eq}}={\left[-\ln(1-a_{w})/A(T+B)\right]}^{1/C}$	(6)
Modified Halsey [37]	$m_{\mathrm{eq}}={\left[-\exp(A+{\mathrm{BT})/\mathrm{lna}}_{w}\right]}^{1/C}$	(7)

Note: m_eq_ is the equilibrium moisture content, decimal (dry-basis), a_w_ is the relative humidity (decimal), T is the absolute temperature (K), A, B, and C are the constant values.

**Table 3:** Volatile organic compounds in para-rubber, bamboo, and eucalyptus wood vinegar.

No.	Compound	Percentage of Total Area		
		Para-Rubber	Bamboo	Eucalyptus
1	Acetic acid	41.34	38.19	31.25
2	Phenol	8.29	7.56	7.12
3	Phenol, 2,6-dimethoxy (Syringol)	7.38	5.53	11.07
4	2-Methoxyphenol (Guaiacol)	2.81	3.27	3.89
5	p-Cresol	1.57	1.72	1.85
Total		61.39	56.27	55.18

**Table 4:** The chemical and physical properties of NR sheets.

Properties of NR Sheets	Types of Coagulating Materials				
	Raw Wood Vinegars				
	Para-Rubber	Bamboo	Eucalyptus	Formic Acid	Acetic Acid
Before drying					
Dirt content (%w/w)	0.052 ± 0.002	0.054 ± 0.003	0.049 ± 0.003	0.043 ± 0.004	0.051 ± 0.002
Volatile content (%w/w)	0.83 ± 0.03	0.85 ± 0.05	0.82 ± 0.05	0.77 ± 0.02	0.83 ± 0.03
Plasticity retention index (PRI)	99.2 ± 2.5	98.7 ± 2.4	102.3 ± 1.8	108.5 ± 2.9	96.8 ± 1.9
Mooney viscosity	52.1 ± 0.4	51.9 ± 0.3	53.2 ± 0.3	54.7 ± 0.2	51.5 ± 0.4
After drying					
Dirt content (%w/w)	0.036 ± 0.003	0.037 ± 0.002	0.034 ± 0.002	0.032 ± 0.003	0.039 ± 0.003
Volatile content (%w/w)	0.45 ± 0.03	0.47 ± 0.02	0.44 ± 0.03	0.41 ± 0.03	0.49 ± 0.02
Plasticity retention index (PRI)	93.9 ± 2.2	94.1 ± 2.7	97.5 ± 2.6	102.9 ± 2.5	92.7 ± 2.2
Mooney viscosity	55.9 ± 0.3	55.2 ± 0.4	56.3 ± 0.4	59.4 ± 0.3	54.8 ± 0.3
Curing time (min)	18.3 ± 0.5	18.9 ± 0.5	17.7 ± 0.8	16.2 ± 0.4	19.5 ± 0.7
Tensile strength at break (MPa)	6.2 ± 0.3	5.9 ± 0.5	4.8 ± 0.5	5.1 ± 0.4	6.4 ± 0.3
Elongation at break (%)	671 ± 8	664 ± 11	625 ± 8	631 ± 12	678 ± 7
300% modulus (MPa)	1.0 ± 0.1	1.0 ± 0.1	1.0 ± 0.1	1.0 ± 0.1	1.0 ± 0.1

**Table 5:** Parameters of the mathematical models for the EMC of NR sheets (wood vinegar derived).

Model	Parameters and Goodness of Fit	Para-Rubber Wood			Bamboo Wood			Eucalyptus Wood		
		Drying Temperature			Drying Temperature			Drying Temperature		
		40 °C	50 °C	60 °C	40 °C	50 °C	60 °C	40 °C	50 °C	60 °C
Oswin	A	0.6589	0.7289	0.3257	1.0252	0.9857	0.9312	1.0345	0.9127	1.0526
	B	0.8261	0.7891	0.9413	0.8957	0.7241	0.8422	1.0031	1.3042	0.8947
	r^2^	0.9772	0.9715	0.9732	0.9722	0.9851	0.9814	0.9927	0.9913	0.9952
	MRD	0.0227	0.0395	0.0412	0.0341	0.0312	0.0435	0.0205	0.0151	0.0177
Smith	A	0.1645	0.1808	0.1082	0.1235	0.1567	0.1051	0.2249	0.2517	0.2135
	B	0.4488	0.5044	0.2273	0.3257	0.2891	0.3125	0.4891	0.4622	0.3985
	r^2^	0.9497	0.9579	0.9078	0.9141	0.9013	0.8976	0.8827	0.9215	0.9351
	MRD	0.0424	0.0327	0.0359	0.0389	0.0412	0.0422	0.0375	0.0326	0.0394
Henderson	A	0.4158	0.4297	0.3267	0.4582	0.4131	0.3594	0.3357	0.3882	0.3801
	B	0.0541	0.0718	0.0821	0.0725	0.0803	0.0863	0.0543	0.0631	0.0615
	r^2^	0.9648	0.9725	0.9528	0.9615	0.9217	0.9516	0.9485	0.9108	0.9253
	MRD	0.0282	0.0325	0.0334	0.0292	0.0304	0.0291	0.0377	0.0364	0.0371
Modified Oswin	A	0.2887	0.3105	0.3321	0.2951	0.3006	0.2574	0.2886	0.3018	0.2135
	B	0.0258	0.0294	0.0315	0.0295	0.0351	0.0312	0.0272	0.0308	0.0367
	C	5.2194	6.0082	5.6842	4.2219	4.3851	5.0214	5.2197	4.8262	4.2159
	r^2^	0.9226	0.9358	0.9412	0.9105	0.9087	0.9521	0.9124	0.9326	0.9572
	MRD	0.0359	0.0397	0.0428	0.0381	0.0395	0.0418	0.0295	0.0316	0.0385
Modified Henderson	A	0.2658	0.2156	0.3247	0.3189	0.4428	0.4816	0.5562	0.5107	0.5113
	B	0.6941	0.3051	0.4518	0.4821	0.3915	0.5237	0.8521	0.8863	0.7642
	C	0.2319	0.3058	0.2816	0.2943	0.3267	0.2641	0.3025	0.3629	0.2856
	r^2^	0.9915	0.9967	0.9928	0.9907	0.9918	0.9952	0.9756	0.9612	0.9705
	MRD	0.0184	0.0201	0.0218	0.0187	0.0176	0.0205	0.0291	0.0316	0.0368
Modified Halsey	A	15.227	11.295	19.251	10.256	9.1275	12.354	2.6422	3.9543	2.8162
	B	9.2185	7.5421	11.265	7.5204	5.3219	6.0058	1.0251	1.6593	1.6945
	C	1.3684	2.3658	1.0064	2.9642	3.0658	3.6419	6.2289	7.2546	5.2157
	r^2^	0.8895	0.9024	0.9117	0.8912	0.9431	0.9357	0.9216	0.8997	0.9176
	MRD	0.0395	0.0375	0.0403	0.0379	0.0357	0.0391	0.0382	0.0408	0.0386

Note: r^2^ is the correlation coefficient and MRD is the mean relative deviation.

**Table 6:** Parameters of the mathematical models for the EMC of NR sheets (commercial acid).

Model	Parameters and Goodness of Fit	Formic Acid			Acetic Acid		
		Drying Temperature			Drying Temperature		
		40 °C	50 °C	60 °C	40 °C	50 °C	60 °C
Oswin	A	0.6857	0.9561	1.0254	0.8899	0.6795	0.2977
	B	0.8624	0.8853	0.8235	0.7955	0.7523	0.9564
	r^2^	0.9922	0.9954	0.9938	0.9721	0.9813	0.9754
	MRD	0.0202	0.0175	0.0194	0.0292	0.0351	0.0372
Smith	A	0.1925	0.2037	0.2185	0.0321	0.0112	0.0437
	B	0.3812	0.4105	0.3992	0.0139	0.0614	0.0842
	r^2^	0.9021	0.9106	0.8973	0.9622	0.9741	0.8579
	MRD	0.0419	0.0367	0.0374	0.0341	0.0253	0.0282
Henderson	A	0.4005	0.3991	0.3462	0.3725	0.3107	0.2975
	B	0.0522	0.0634	0.0593	0.0219	0.0261	0.0189
	r^2^	0.9027	0.9564	0.9629	0.9753	0.9618	0.9827
	MRD	0.0355	0.0311	0.0395	0.0317	0.0398	0.0356
Modified Oswin	A	0.2612	0.2591	0.2982	0.1776	0.2188	0.2745
	B	0.0259	0.0286	0.0317	0.0086	0.0067	0.0132
	C	5.0068	5.8125	5.2953	4.0841	0.4188	8.6156
	r^2^	0.9615	0.9214	0.9362	0.9695	0.9525	0.9462
	MRD	0.0327	0.0371	0.0405	0.0271	0.0243	0.0315
Modified Henderson	A	0.4921	0.4527	0.4118	0.0513	0.0193	0.2298
	B	0.9216	0.8611	0.9028	0.6705	0.4438	0.2007
	C	0.2785	0.3192	0.3109	0.4043	0.1469	0.0482
	r^2^	0.9837	0.9724	0.9778	0.9902	0.9918	0.9965
	MRD	0.0352	0.0271	0.0344	0.0152	0.0211	0.0193
Modified Halsey	A	7.2158	4.3269	6.3251	1.3382	4.7023	28.761
	B	6.3142	7.2261	8.1165	0.7547	6.4395	13.707
	C	1.9967	2.3154	1.2635	7.1962	1.0211	0.6212
	r^2^	0.8722	0.8753	0.8931	0.9066	0.9242	0.8748
	MRD	0.0425	0.0379	0.0418	0.0271	0.0415	0.0432

Note: r^2^ is the correlation coefficient and MRD is the mean relative deviation.

## Data Availability

The original contributions presented in this study are included in the article. Further inquiries can be directed to the corresponding author.
